# Compliance for decontamination of bedside computer keyboards on an ICU

**DOI:** 10.1186/cc10685

**Published:** 2012-03-20

**Authors:** A Disney, K Sim

**Affiliations:** 1 St Helens & Knowsley NHS Trust, Prescot, UK

## Introduction

An audit to assess the compliance of decontamination of bedside computer keyboards by medical staff on an ICU. The topic was used for audit as previously identified by the hospital infection control department as the worst-performing area on 'clean trace' scoring, and had been a regular topic of discussion between members of the medical team. Bedside keyboards automatically emit an alarm sound and small flashing LED light if cleaning is required prior to use. Discussion and hypothesis on reasons for rates of compliance follow this, including social/human factors that are barriers to compliance with infection control measures.

## Methods

Observations and recording of medical staff and their compliance of cleaning bedside keyboards when required during a morning consultant-led ward round over a 10-day period. Further observations were taken at two other points in time during each day over the same time period. Observations were conducted by the author. Medical staff were informed at the beginning of the audit that this behaviour was being observed and staff were reminded of the principles of infection control, specifically maintaining clean IT equipment. A standard of 100% compliance for cleaning the keyboard appropriately when required was set for audit purposes.

## Results

Total number of patient consultations during morning ward round *n *= 99; total number of occasions a keyboard used *n *= 40; total number of times a keyboard used and keyboard required cleaning *n *= 37; keyboard used and cleaned when required prior to use *n *= 5. In total, a rate of compliance at 14% for cleaning keyboards when appropriate. Further observations over the same time period showed that keyboards were indicating they required cleaning on 96% of occasions observed.

## Conclusion

The rate of compliance for cleaning bedside keyboards was 14%, below the standard set of 100%. This may be due to several factors, including lack of education and resistance to changes in behaviour which have previously been described in various models of human behaviour such as the theory for planned behaviour. This may reflect compliance with other areas of infection control and have a detrimental effect on patient safety and health. This may also become a significant issue in the future if compliance rates remain low as more IT and touchscreen equipment is employed in ICUs. The ICU has adjusted the induction programme for new medical staff to include education on infection control measures with bedside IT equipment.

